# Safety, mucosal and systemic immunopotency of an aerosolized adenovirus-vectored vaccine against SARS-CoV-2 in rhesus macaques

**DOI:** 10.1080/22221751.2022.2030199

**Published:** 2022-01-29

**Authors:** Fang Xu, Shipo Wu, Linan Yi, Shaodan Peng, Fan Wang, Weixue Si, Lihua Hou, Tao Zhu

**Affiliations:** a CanSino Biologics, Tianjin, People’s Republic of China; b Institute of Biotechnology, Academy of Military Medical Sciences, Beijing, People’s Republic of China

**Keywords:** COVID-19, SARS-CoV-2 vaccine, inhalation, aerosol, safety, mucosal immunity, immunoglobulin A

## Abstract

Mucosal immunity provides a potential for preventing initial infection and stopping subsequent transmission of SARS-CoV-2. Here, we examined the safety and immunogenicity of a replication-defective adenovirus type-5 vectored vaccine (Ad5-nCov) encoding SARS-CoV-2 spike protein delivered by nebulization inhalation in rhesus macaques. The vaccine-associated clinical pathology and toxicity were not observed in the NHP model. The extensive safety study indicated that Ad5-nCoV was mainly confined to the organs related to respiratory system and was rapidly cleared away from the system. Our results showed that Ad5-nCoV delivered by inhalation robustly elicited both systematic and mucosal immune responses against SARS-nCoV-2 and variants. Thus, Ad5-nCoV inhalation may provide an effective, safe and non-invasive vaccination strategy for the control of SARS-CoV-2.

## Introduction

Severe acute respiratory syndrome coronavirus-2 (SARS-CoV-2), also known as COVID-19, has caused one of the biggest global pandemics and crises throughout history. As of 5 November 2021, more than 248 million confirmed COVID-19 cases including 5 million deaths were reported to World Health Organization (WHO). The global scientific community, including the academic research and pharmaceutical industry, has endeavoured to accelerate vaccine development to control COVID-19 spread. The latest data indicate that 18 COVID-19 vaccines currently in global use include mRNA vaccines, recombinant adenovirus vectored vaccines, inactivated vaccines, and subunit vaccines [1]. So far, 50.9% of the world population has received at least one dose of a COVID-19 vaccine. The massive vaccination has provided an effective tool for the global control of the COVID-19 pandemic.

Most SARS-CoV-2 vaccines, either in current application or in clinical trials, have focused on systemic immunization by an intramuscular administration to achieve sufficient protection against SARS-CoV-2 [2]. However, the vaccine-induced mucosal immunity for SARS-CoV-2 prevention remains unexplored. The virus initiates the infection through primarily attaching mucosal surfaces of the respiratory tracts via binding to the angiotensin-converting enzyme 2 (ACE-2) receptor [3,4], indicating that the respiratory mucosae explicitly acts as the frontline of the immune system in response to viruses. Consequently, mucosal immunity has the potential to provide the robust defense to prevent initial infection and subsequent transmission, which has been exemplified by recent studies in animal models on mucosal vaccines of SARS-CoV-2 that showed that mucosal immunity combining with systematic immunity can fully protect against SARS-CoV-2 [5–7].

Ad5-nCoV, which has been developed by CanSino Biologics Inc. and Beijing Institute of Biotechnology, is a replication-defective adenovirus type-5 vectored vaccine encoding the SARS-CoV-2 spike protein [8]. Ad5-nCoV has demonstrated a good safety and high efficacy against severe disease in clinical trials and massive vaccination campaigns [9]. Here, we, for the first time, reported the safety, mucosal and systematic immunogenicity of aerosolized Ad5-nCoV vaccine by means of nebulization inhalation that was firstly utilized for SARS-CoV-2 vaccine delivery in rhesus macaques.

## Nebulization inhalation administration

The delivery route of Ad5-nCoV mucosal vaccine is pivotal for effectively delivering vaccine to the mucosal surface and eliciting both strong local and systematic immunity. To find out the optimal mucosal administration, the macaques were vaccinated with Ad5-nCoV at the same dose through intranasal, nebulization inhalation and intratracheal aerosol administration, respectively. Spike-specific antibody responses have been proposed to be the crucial protective mechanisms for COVID-19 vaccine strategies. We measured the S-specific IgG and S-RBD-specific IgA titres in serum by ELISA. Both elicited IgG and IgA responses were significantly stronger in the animal group of nebulization inhalation compared to the other two administration routes. In addition, inhaled Ad5-nCoV provided around 6-month prolonged antibodies persistence, whereas the humoral responses in other groups declined rapidly after 10 weeks after boost immunization (see Supplementary Figure 1). The primary data strongly demonstrated that nebulization inhalation can be an optimal administration route for the subsequent study of respiratory mucosal vaccine of Ad5-nCoV.

## Safety profile

Pre-clinical studies were performed in rhesus macaques to determine the extensive safety profile of the biodistribution and clearance of Ad5-nCoV in the system, and to screen for potential vaccine-related toxicities. Two groups of rhesus macaques (*n* = 10, 5 per gender) at 3–6 years of age were immunized by nebulization inhalation (Aerogen ® Solo system) with a low dosage of 2.5 × 10^10^ viral particles (vp) and a high dosage of 1.5 × 10^11^ vp per animal, respectively. The booster vaccine was administrated at 14 and 28 days post-prime immunization (see Supplementary Figure 2). Animals were monitored daily based on the following criteria: general appearance, mortality, morbidity, discharge, respiration, feces and urine appearance, appetite, and activity. In addition, the treatment-related clinical pathology and toxicity were studied. There were no abnormal clinical signs noted. The parameters of clinical pathology and systematic toxicity appeared to have not been negatively impacted by vaccination.

**Figure 1.**
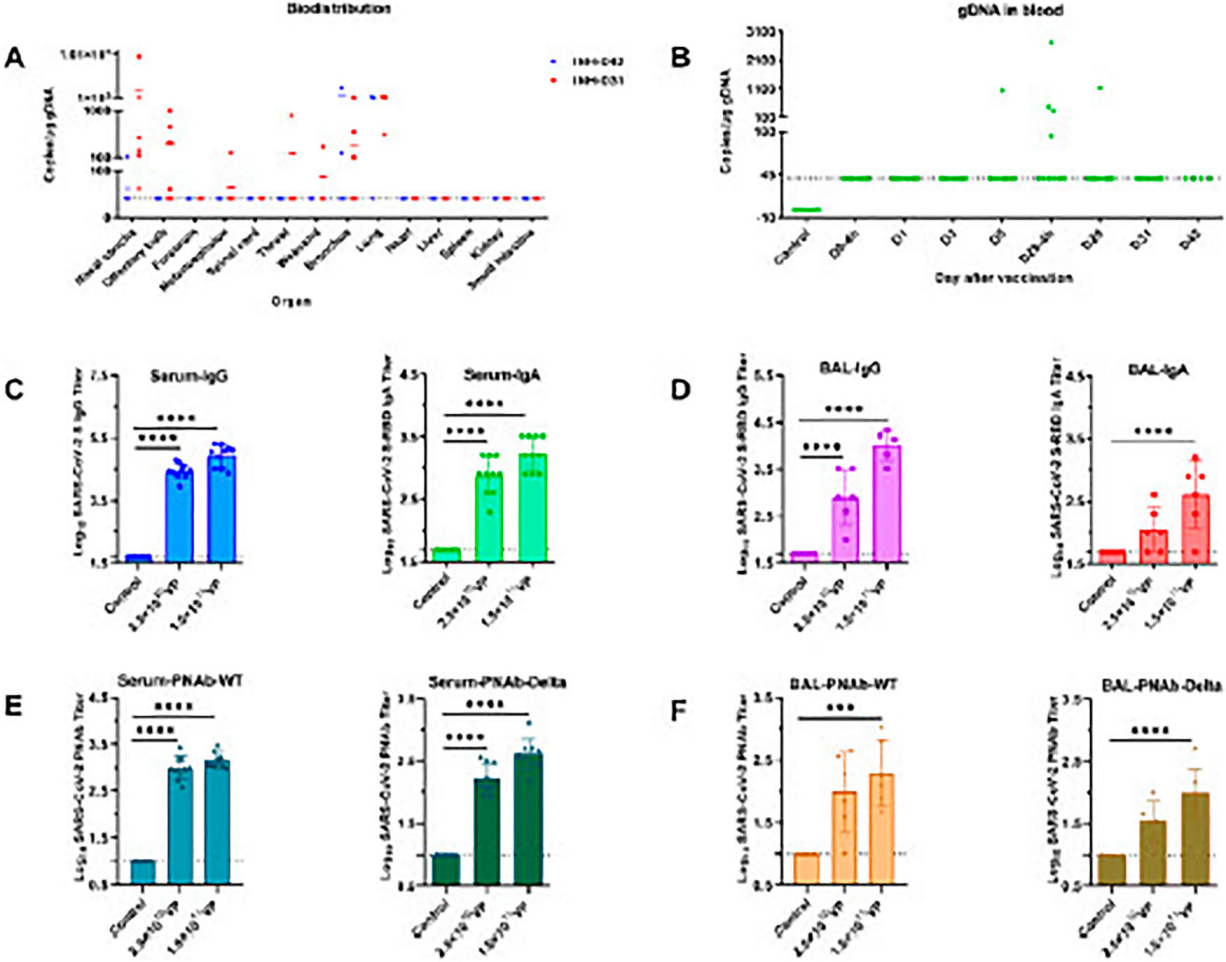
Safety and immunogenicity of aerosol Ad5-nCoV. (A) Biodistribution of Ad5-nCoV in the tested organs collected on 31 and 42 DPI. The viral genome in the tissues was assessed with qRT-PCR. (B) Ad5-nCoV presence in the blood at the indicated time points. (C and D) S-specific humoral immune responses in serum and bronchoalveolar lavage. (E and F) Pseudovirus neutralization antibody (PNAb) titres for SARS-CoV-2 WT or Delta variant were elevated in blood and BLA, respectively. ****P < 0.0001, ***P < 0.001.

The organs were collected at 31 days post immunization (DPI) and 42 DPI for biodistribution investigation using Quantitative Rea-time-PCR (Figure 1A). In a general biodistribution pattern, Ad5-nCoV remained predominant at the sites of respiratory tract and organs, including throat, nasal concha, bronchus and lung, and exclusively biodistributed to the olfactory bulb, metencephalon and weasand shortly after boost immunization. The absence of Ad5-nCoV DNA in most of the tested organs and the rapid decline of Ad5 copy number in nasal concha and bronchus at 42 DPI indicated that Ad5-nCoV rapidly cleared away from the system over time, though ∼10^11^ vp were inoculated. In addition, Ad5-nCoV DNA in the blood were only detected in limited animals shortly after immunization, which indicated that Ad5-nCoV is mainly confined to the organs related to respiratory system instead of trafficking to other important organs via blood (Figure 1B). Taken together, the safety data demonstrate the pattern of limited biodistribution and extensive systematic clearance of Ad5-nCoV.

## Systematic and mucosal immune responses

To evaluate the immunogenicity of aerosol Ad5-nCoV vaccine in rhesus macaques, serum samples were collected at the indicated days following vaccine administration. At 14 days post second immunization, robust systematic antigen-specific antibody responses including IgG and IgA, were elicited in all vaccinated animals, which demonstrated strong systematic immune response in a dose-dependent manner (Figure 1C).

Mucosal immunization is known to stimulate mucosal IgA antibodies, which can capture and neutralize respiratory pathogens on the mucosal surfaces, thus providing the first line of defense against infection. Therefore, we collected the bronchoalveolar lavage (BAL) and assessed the mucosal immunity by measuring S-RBD specific IgA titre. As shown in Figure 1(D), 3 days after the last immunization, robust mucosal S-RBD specific IgA, including IgG responses, were detected in BAL. Notably, the high dosage group exhibited about 10-fold higher mucosal IgA and IgG titres compared to the low dosage group.

Vaccine-elicited neutralizing antibody responses in serum and BAL against SARS-CoV-2 variants were evaluated with a pseudovirus-based neutralization assay as described previously [10]. Pseudoviruses-specific neutralizing antibodies (PNAb) were significantly stimulated after the last immunization and detectable in all vaccinated animals. However, a slight decline of PNAb titres against pseudovirus of Delta variant in vaccinated animals was observed (Figure 1, E and F). Taken together, our findings revealed that inhaled aerosol Ad5-nCoV vaccine induced the potent S-specific Ab responses in blood and BAL, including the strong neutralizing capacity, which indicated the robust systematic and mucosal immune responses were elicited with inhaled aerosol Ad5-nCoV vaccine.

## Cellular immune responses

We also evaluated the subsets of S-specific memory CD4+ (mCD4+) and memory CD8+ (mCD8+) T-cell responses in the peripheral blood mononuclear cells (PBMCs) of the vaccinated animals. The data demonstrated that strong IFNγ and TNF responses in both mCD4+ and mCD8+ cells 2 weeks post the second vaccination were induced (see Supplementary Figure 3). Intracellular cytokine staining confirmed that aerosol Ad5-nCoV elicited a high frequency of mCD4+ T cells that produced IFNγ, TNF and IL-2 but barely IL-4 expression in mCD4+ T cells (see Supplementary Figure 3A). Similarly, the robust responses of IFNγ and TNF but IL2 and IL4, were stimulated in mCD8+ T cells, which indicates a Th1-biased response (see Supplementary Figure 3B).

## Conclusion

In this study, we have demonstrated that inhaled aerosol Ad5-nCoV is a safe and immunogenic mucosal vaccine against SARS-CoV-2 and variants in NHPs. Given this aerosol vaccine-induced strong local and systematic immunity, it may facilitate the prevention and mitigation of COVID-19 by providing comprehensive protection from virus infection and transmission. Besides its high efficacy, the inhalation delivery remains a simple, non-invasive and cost-effective vaccine administration, which may support an attractive and practical alternative to intramuscular application for control of COVID-19.

## Supplementary Material

Supplemental Material
