# Supplementary material for: Safety, mucosal and systemic immunopotency of an aerosolized adenovirus-vectored vaccine against SARS-CoV-2 in rhesus macaques
Source: Emerg Microbes Infect. 2022 Jan 29;11(1):438–41. doi: 10.1080/22221751.2022.2030199 (PMC8803102; doi:10.1080/22221751.2022.2030199)
Supplement: Supplemental Material [file TEMI_A_2030199_SM5753.zip › Suppl Files/EMI Supplementary materials CS 20220111 clean.docx]

#
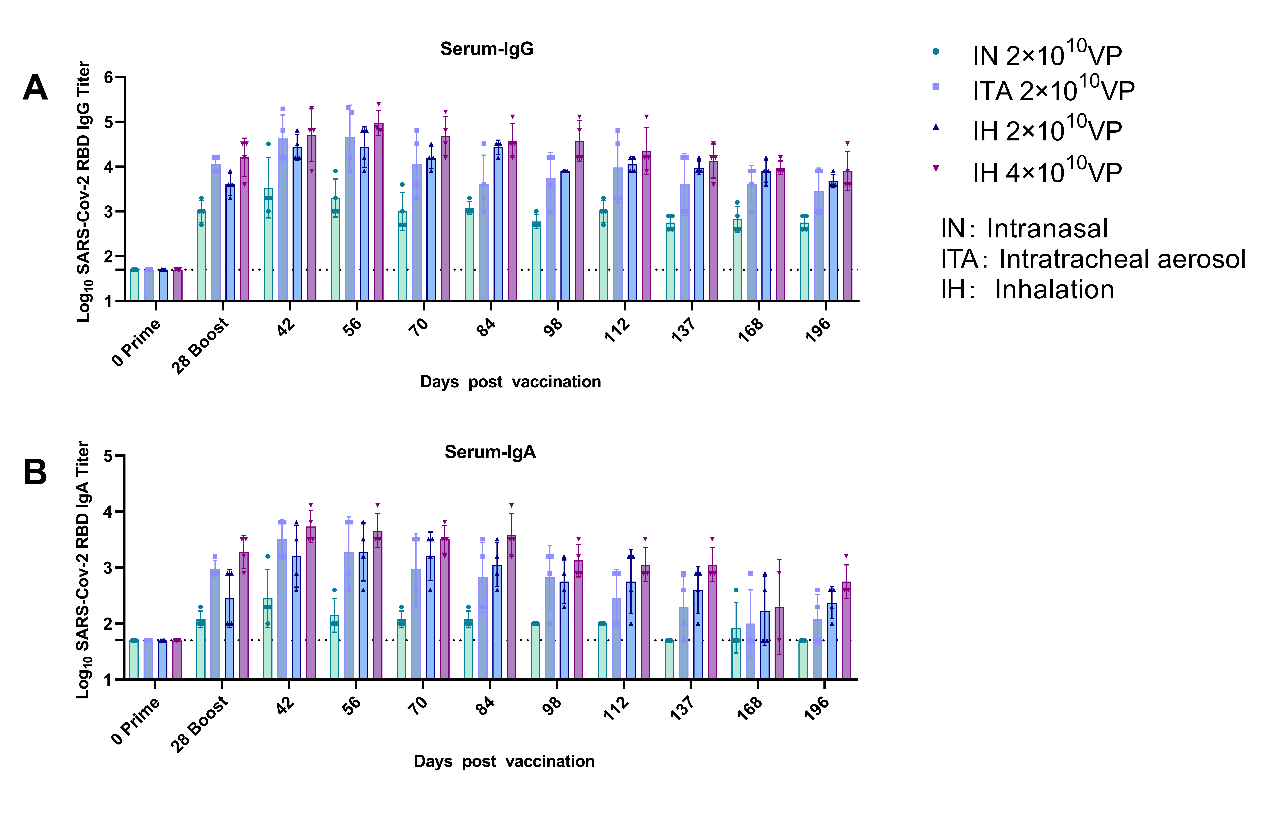
Supplementary material


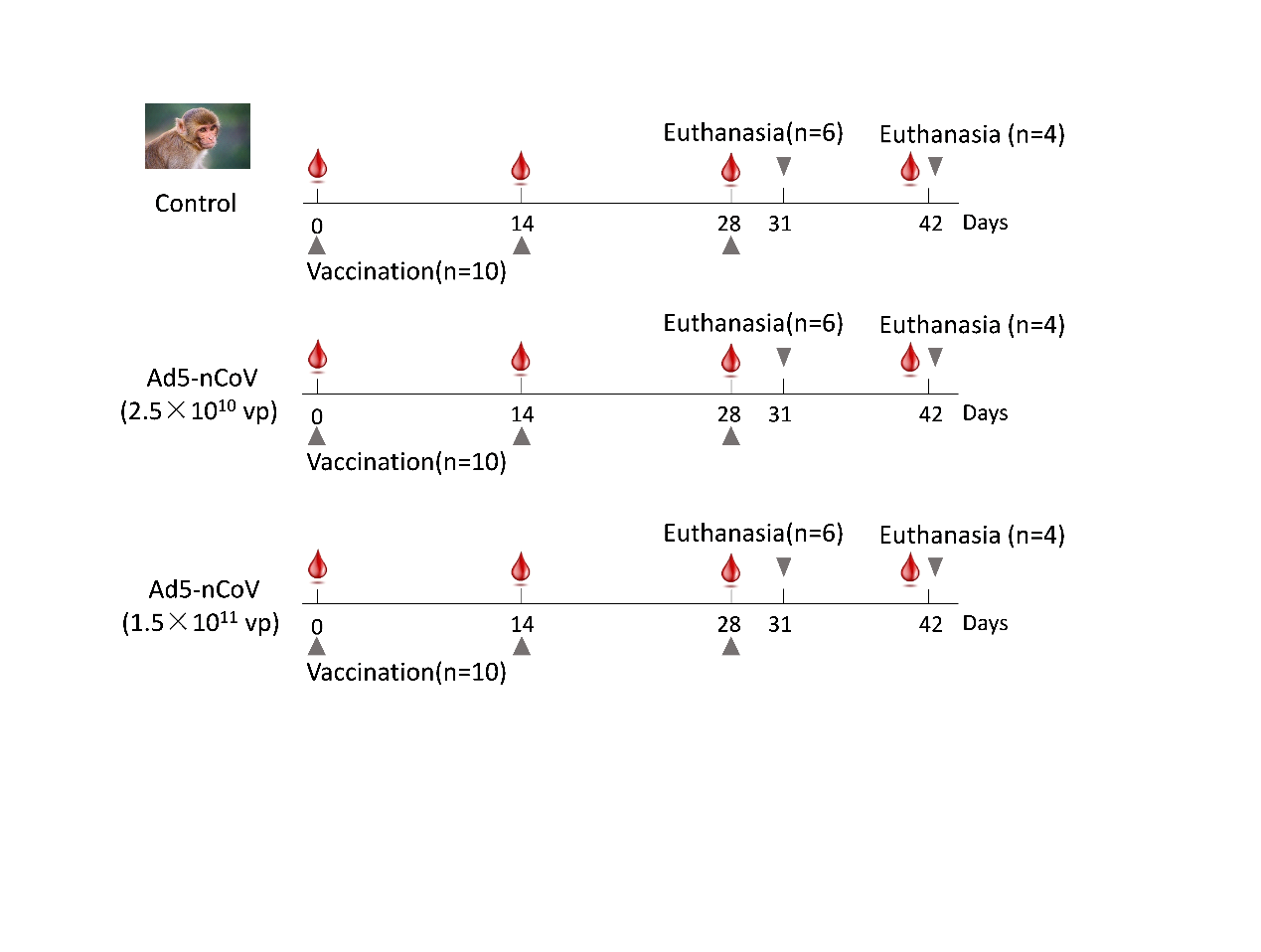
**Supplementary Figure 1.** The immunogenicity of Ad5-nCoV in the animals after administration via intranasal, intratracheal aerosol or inhalation route. (A) S-RBD specific IgG antibody responses in serum. (B) S-RBD specific IgA antibody responses in serum.

**Supplementary Figure 2.** Schematic diagram of immunization protocol. 3-6 years old rhesus macaques (n=10) were vaccinated with Ad5-nCoV via inhalation at day 0, day 14 and day 28 using the indicated dosage. The red blood drop symbols indicate the time points for serum sample collection.

**Supplementary Figure**

**3.** S-specific mCD4+ and mCD8+ T cell responses in PBMCs two weeks after the second vaccination. (A) S-specific IFNγ, TNF, IL2 and IL4 mCD4+ T cell responses (B) S-specific IFNγ, TNF, IL2 and IL4 mCD8+ T cell responses.

**** P<0.0001, *** P<0.001, **P<0.01, *P<0.05
