# Supplementary figures and images for: Safety, mucosal and systemic immunopotency of an aerosolized adenovirus-vectored vaccine against SARS-CoV-2 in rhesus macaques
Source: Emerg Microbes Infect. 2022 Jan 29;11(1):438–41. doi: 10.1080/22221751.2022.2030199 (PMC8803102; doi:10.1080/22221751.2022.2030199)

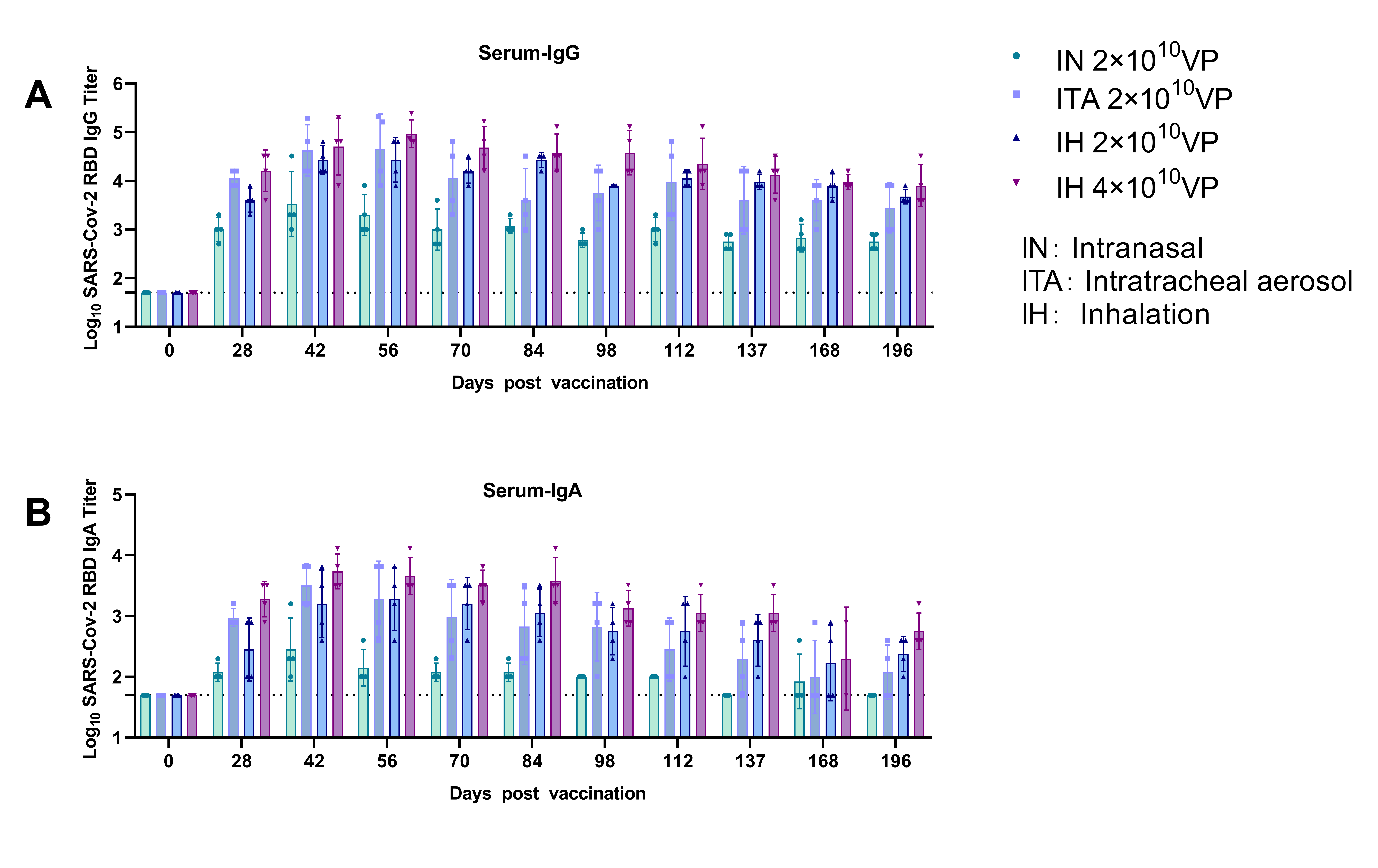

Supplement: Supplemental Material [file TEMI_A_2030199_SM5753.zip › Suppl Files/Supplementary Figure 1.tif]
